# Aggregation-Induced Emission (AIE) Probe-Labeled Nanotheranostics: A Mini-Review

**DOI:** 10.3390/ph19060902

**Published:** 2026-06-06

**Authors:** Yilin Ma, Yingying Li, Chuanbin Wu, Yao Yang, Xin Pan, Zhengwei Huang

**Affiliations:** 1 International School, Jinan University, Guangzhou 510632, China; mayilin0815@163.com; 2 Anhui Engineering Technology Research Center of Biochemical Pharmaceutical, Bengbu Medical University, Bengbu 233030, China; yingyingli@bbmu.edu.cn; 3 State Key Laboratory of Bioactive Molecules and Drugability Assessment, Guangdong Basic Research Center of Excellence for Natural Bioactive Molecules and Discovery of Innovative Drugs, College of Pharmacy, Jinan University, Guangzhou 511436, China; chuanbinwu@jnu.edu.cn; 4 Institute of Pharmaceutics, School of Pharmaceutical Sciences (Shenzhen), Sun Yat-Sen University, Shenzhen 518107, China; 5 School of Pharmaceutical Sciences, Sun Yat-Sen University, Guangzhou 510006, China; panxin2@mail.sysu.edu.cn

**Keywords:** aggregation induced emission (AIE), nanotheranostics, AIEgens, bioimaging, drug delivery, cancer theranostics

## Abstract

Nanotheranostics integrate theranostic functions onto a single nanoscale platform, and have become a new approach in precision medicine. Nanotheranostics rely on probes. However, traditional fluorescent probes often exhibit aggregation-caused quenching (ACQ) when loaded at high concentrations onto nanocarriers, severely limiting their imaging performance. Aggregation-induced emission agents (AIEgens) offer a solution to this long-standing problem through their ability to enhance fluorescence during aggregation. This mini-review systematically outlines nanotheranostic systems based on aggregation-induced emission (AIE). We first introduce the basic mechanism of AIE (the limitation of molecular internal motion) and its advantages over traditional fluorescent probes. Then, we discuss the design strategies of AIE nanoprobes according to the types of nanocarriers (including liposomes, polymer nanoparticles, and self-assembling systems). Additionally, we emphasize the disease-specific AIE nanotheranostic designs tailored for pathological microenvironments such as tumors, neurodegenerative diseases, and inflammatory diseases. Finally, we conduct an in-depth analysis of the current challenges hindering clinical translation, and propose future AIE nanotheranostic technologies applicable to clinical practice and the direction for personalized medicine.

## 1. Introduction

Nanomaterials are defined as materials composed of nanoparticles, where at least 50% of the nanoparticles have an external size ranging from 1 to 100 nm. Their small size not only allows for more surface functionality within a given volume, but also leads to different physical properties in many aspects (including electronic, optical, and magnetic characteristics) compared to their bulk counterparts [1]. Firstly, as the material size decreases to the nanoscale, its specific surface area increases dramatically. For instance, when an iron cube is reduced from 1 cm^3^ to 10 nm^3^, the proportion of surface atoms increases by approximately 10%; upon further reduction to 1 nm^3^, virtually all atoms reside on the surface. Correspondingly, for an equivalent total volume, the total surface area expands from 3.6 cm^2^ for a 0.77 cm diameter particle to 2.8 × 10^7^ cm^2^ for a 1 nm diameter particle. This dramatic amplification of surface-to-volume ratio underpins many of the unique physicochemical properties of nanomaterials [2]. Secondly, when the material size approaches or is smaller than certain characteristic physical scales (such as exciton Bohr radius and average electron free path), physical phenomena specific to the nanoscale, such as quantum confinement effects and surface plasmon resonance, begin to manifest, resulting in different properties in optics, electricity, magnetism, thermodynamics, etc., compared to their macroscopic bulk materials. Beyond these fundamental physical changes, the reduced dimensions also deeply change the biological interactions of materials. For example, the bulk polymeric materials do not typically enter cells, but at the nanoscale, their cellular uptake behavior becomes extremely size-dependent [3]. A systematic fluorescence microscopy study showed that fluorescent nanoparticles with diameters below 10 nm accumulate at the plasma membrane before internalization, while larger nanoparticles of 100 nm are directly internalized without prior membrane accumulation, independently of their surface charge. In this different size-dependent uptake mechanism, even a few tens of nanometers dictate the route of cellular entry. Therefore, the nanoscale transition of particles not only changes physical properties, but also fundamentally changes the material’s biological identity and interactions [4].

**Figure 1:**
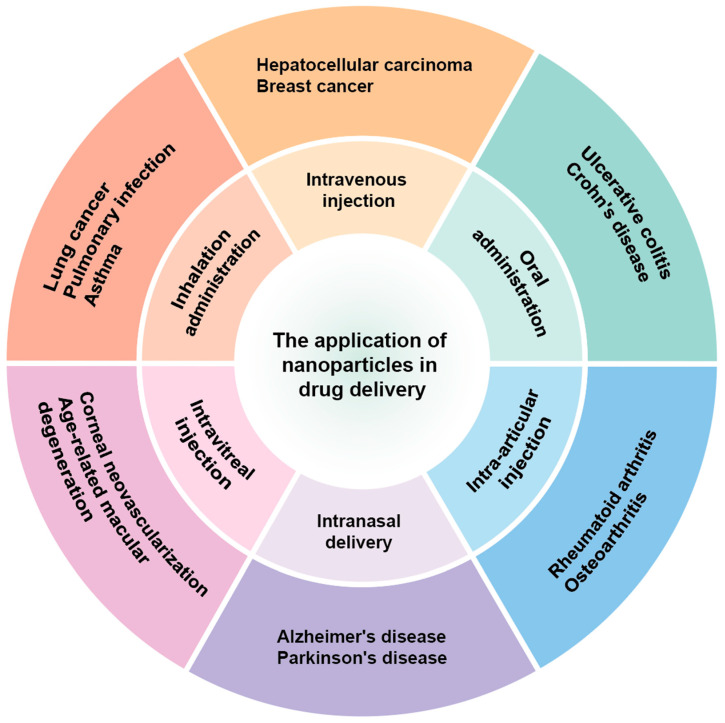
This figure illustrates the classification of some applications of nanomaterials in six administration routes [5,6,7,8].

Besides the above several different nano-sized attributes, versatile nanomedicines have also been designed and developed for disease diagnosis and therapy. The diagnostic and therapeutic applications of nanomaterials can be engineered by diverse administration routes, which in turn have different advantages for targeting the disease sites. As shown in Figure 1, six representative delivery strategies with typical therapeutic meanings are shown: intravenous injection for system delivery for hepatocellular carcinoma and breast cancer; oral for ulcerative colitis and Crohn’s disease to treat in the colon; intra-articular injection for rheumatoid arthritis and osteoarthritis; intranasal delivery to bypass the blood–brain barrier (BBB) of Alzheimer’s and Parkinson’s disease; intravitreal injection for ocular conditions, including corneal neovascularization and age-related macular degeneration; and inhalation for pulmonary indications, for example, lung cancer (hopefully), pulmonary infection and asthma. These administration routes show the versatility of the nanocarrier platform in achieving the site-specific accumulation and controlled drug release for a wide variety of diseases. All of the above content holds great research and application value. Detailed content can be found in Figure 1.

In recent years, under the further development of nanoscience and nanotechnology, the concept of nanotheranostics emerged. It involves integrating therapeutic agents and imaging agents into a single nanocarrier, forming a multifunctional platform that combines diagnosis and treatment, enabling non-invasive diagnosis of diseases; real-time monitoring of treatment, prediction and evaluation of treatment responses; and thus promotion of personalized medicine [9]. By combining multiple modalities of diagnosis such as fluorescence imaging, magnetic resonance imaging (MRI), positron emission tomography (PET), and computed tomography (CT), and using therapeutic agents in a synergistic manner, diagnosis and treatment can be integrated in the same time and space [9]. As the current research hotspots of nanotheranostics are associated with cancer therapy, the abovementioned therapeutic agents are mainly referred to as drugs for chemotherapy, radiotherapy, photothermal therapy, gene therapy, and immunotherapy. Of note, fluorescence imaging has significant advantages over other diagnostic methods (such as MRI, PET, and CT), and is highly suitable for integration into the nanotherapy diagnostic platform [10]. Fluorescence imaging can achieve real-time, high-resolution visualization of biological processes and has excellent spatiotemporal resolution [11,12]. Moreover, compared to PET, fluorescence imaging does not involve ionizing radiation and can perform repeated, non-invasive monitoring without the need to synthesize radioactive markers and decay limitations [10]. Most importantly, the molecular diversity of fluorescent probes enables their specific targeting through chemical modification [13]. Given these practical advantages, fluorescent probes have become the core of nanotheranostic research. Therefore, this review will mainly focus on fluorescent probes and consider them as the diagnostic core of the nanotheranostic system.

Compared to traditional diagnosis and treatment methods, integration by nanotherapeutics demonstrates significant advantages:

(1) Promoting personalized/precision medicine: These systems can monitor the distribution of drugs in the body, their accumulation at the target site, and their release status in real time and non-invasively during the treatment process [14]. However, it is important to note that such monitoring remains currently unfeasible in humans, especially for AIE-based probes, for at least two reasons: first, because the AIE signal is by essence non-linear with respect to the concentration of molecules or particles; second, because due to the size of the human body (typically 20 cm thickness), attenuation effects are major and cannot be extrapolated from small animal data. Without waiting for traditional endpoint indicators (such as tumor shrinkage), it can provide feedback on the efficacy early and provide dynamic monitoring and intervention, and can promptly adjust the treatment plan [15]. Based on the real-time imaging and biological feedback of each patient, we can tailor and optimize the treatment dosage and scheme [14]. Additionally, by using diagnostic images, we can predict which patients may have a good response to treatment (based on the enrichment degree of the nanomedicine in the lesion) [16]. It enables patient stratification, avoids using ineffective and toxic treatment regimens for non-responders, and optimizes medical resources [14]. Thus, “using the right drug at the right time for the right patient” can be truly achieved, maximizing efficacy and minimizing toxic side effects [14,15].

(2) Enhancing targeting and efficacy, and reducing toxicity: Utilizing the EPR effect of the nanocarriers themselves (passive targeting) and the surface-modified ligands (active targeting), sensors can achieve the high-sensitivity detection of extremely low-concentration biomarkers [17], and meanwhile the drug accumulation at the lesion site can be increased [16]. Therefore, the ability for early diagnosis can be improved and the therapeutic effect enhanced, while reducing damage to healthy tissues and minimizing systemic toxicity [18].

(3) Multifunctional integrated platform with functional combination potential: The fluorescence-based platform can be compatible with multiple imaging modes (MRI, ultrasound, photoacoustic imaging, etc.) and multiple treatment methods (chemotherapy, photothermal therapy, photodynamic therapy, gene therapy, etc.) [15,18]. It has high flexibility and can be designed as the most suitable integrated diagnosis and treatment strategy according to the disease type and treatment requirements [19]. Functionalized nanoparticles can also regulate the immune response and alleviate inflammation, and be developed into nanovaccines [17]. In addition, multivalent nanomaterials can strongly bind to multiple pathogenic agents, suitable for wide-spectrum detection [17].

By virtue of the above strengths, nanotheranostics have been comprehensively investigated nowadays. For example, Zhang et al. developed a polaron engineering strategy to tune the optical properties of carbon quantum dots (CQDs) for NIR-II fluorescence imaging and photothermal therapy. By introducing diverse defects into CQDs, lattice distortions induce polaron formation that enables NIR-II absorption for both fluorescence imaging and photothermal therapy. These CQDs were successfully applied to in vivo tumor imaging and photothermal ablation, demonstrating the potential of nanomaterials as non-AIE fluorescent probes for integrated cancer diagnosis and therapy [20]. In another study, a nanotheranostic platform (FN@P-GA) was constructed by forming J-aggregates of multi-group cyanine dyes, achieving an NIR-II fluorescence emission with a high photothermal conversion efficiency of 57.6%. Owing to its ability to effectively down-regulate HSP90 expression and reduce cellular thermoresistance, FN@P-GA successfully achieved an NIR-II fluorescence imaging-guided mild photothermal therapy under 1064 nm laser irradiation at a low power density (0.3 W/cm^2^). In tumor-bearing mouse models, this cyanine dye-based nanoplatform demonstrated potent anti-tumor effects while minimizing thermal damage to surrounding healthy tissues. This exemplifies the utility of conventional organic fluorophores for deep-tissue imaging and controlled phototherapy [21]. Furthermore, Yin et al. developed a ratiometric NIR-II fluorescent organic nanoprobe, BTz-IC@IR1061, by co-doping the small-molecule photosensitizer BTz-IC with the commercial dye IR1061. This nanoprobe responds specifically to hypochlorite (HClO) within tumors, enabling ratiometric fluorescence changes that simultaneously activate photodynamic therapy (PDT). The presence of HClO selectively restores BTz-IC fluorescence and ROS generation while degrading IR1061, allowing real-time monitoring of PDT activation through ratiometric NIR-II fluorescence imaging. In vivo studies demonstrated effective tumor targeting and significant tumor growth inhibition via tumor-activated PDT. These findings highlight the potential of nanotheranostics in disease management [22].

**Figure 2:**
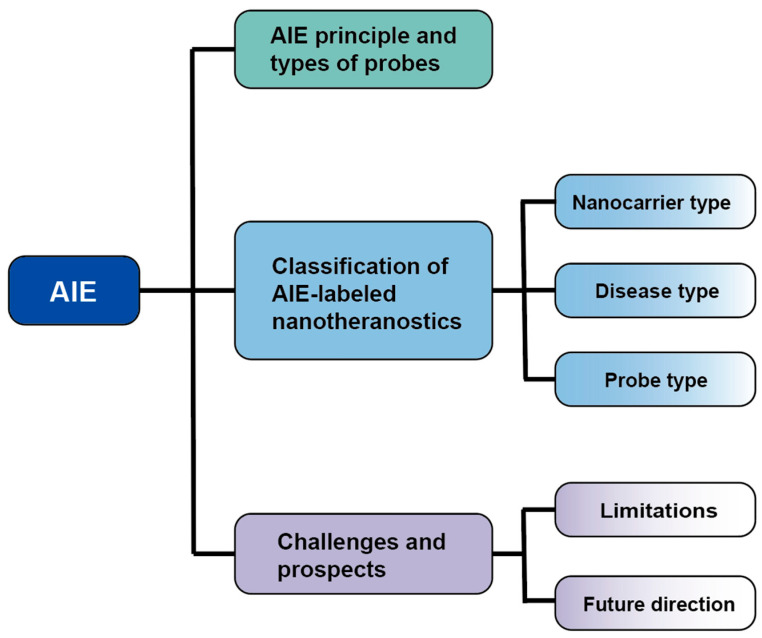
Schematic outline of this mini-review.

Of note, light has been shed on nanotheranostic systems containing fluorescence probes with special functionalities. In recent years, fluorescent probes based on AIE mechanisms have become a burgeoning momentum for nanotheranostics, which introduces new insights into this field. As a characteristic category of fluorescence probes, AIEgens possess outstanding attributes for theranostics, which will be demonstrated below. This mini-review article aimed to present the latest research progress of nanotheranostic systems based on AIE fluorescence probes. The subsequent content will first introduce the basic principles of AIE materials, then elaborate on the construction strategies of AIE-based nanotheranostic systems, and finally discuss the challenges in this field and outline its future development directions. The logic flow is depicted in Figure 2.

## 2. AIE

### 2.1. Discovery of AIE Phenomenon

The discovery of the AIE phenomenon has overturned traditional understanding in the field of luminescence for over sixty years, opening up a new direction for the research of organic luminescent materials. In the mid-20th century, German scientists first discovered and described the “ACQ” phenomenon: many traditional fluorescent groups (such as fluorescein, rhodamine, and azo dyes) can efficiently emit fluorescence in dilute solutions, but as the concentration increases or aggregation occurs, their fluorescent emission intensity significantly weakens or even completely quenches [23]. The physical essence of this phenomenon lies in the strong π-π stacking interaction between aromatic rings in the aggregated state, which promotes the formation of excited-state complexes, resulting in the dissipation of excitation energy through non-radiative pathways. Due to the fact that the luminescent materials are mostly in an aggregated state in practical applications (such as solid-state films, biological imaging, and optoelectronic devices), the ACQ effect has become a major bottleneck restricting the development of traditional fluorescent materials [23].

In 2001, the team led by Academician Benzhong Tang was studying silole derivatives when they observed a phenomenon completely opposite to that of ACQ: these molecules hardly emitted fluorescence in dilute solutions in good solvents (such as tetrahydrofuran), but when they were induced to aggregate by adding poor solvents (water), the fluorescence intensity significantly increased, reaching 10^1^~10^2^ fold [24]. This anomalous phenomenon was initially regarded as an experimental error, but after repeated verification, the team confirmed its scientific authenticity and creatively proposed the concept of AIE, thus pioneering a new field of luminescent materials led by Chinese scientists [25]. Table 1 summarizes the differences between ACQ and AIE.

After the discovery of the AIE phenomenon, researchers proposed various possible mechanism hypotheses, including J-aggregation, conformational flattening, E/Z isomerization, twisted intramolecular charge transfer (TICT), etc. [26]. However, most of them were only applicable to specific systems. Through systematic experiments and theoretical simulations, the restriction of intramolecular motions (RIM) is now confirmed as the universal mechanism of the AIE phenomenon [27,28].

### 2.2. RIM Mechanisms

Restriction of internal rotational (RIR) and restriction of internal vibrational (RIV) are unified into a more universal mechanism framework of RIM [27,28,29]. The RIM mechanism serves as the overarching theoretical foundation for the AIE phenomenon, and the sub-theories RIR and RIV are two distinct but complementary mechanisms. Which mechanism dominates depends on whether the energy dissipation in dilute solution is primarily governed by the rotational or vibrational degrees of freedom of the molecular framework. The following sections detail these two pathways.

(1) RIR

For typical AIE molecules such as TPE, their molecular structure is composed of multiple freely rotatable aromatic rings connected by single bonds [30]. In dilute solutions, these aromatic rings undergo active intramolecular rotations, constantly consuming the excitation energy and dissipating it through non-radiative pathways, resulting in weak fluorescence. When the molecules enter the aggregated state, due to spatial steric hindrance and intermolecular interactions, intramolecular rotations are strongly inhibited, and the non-radiative energy dissipation pathways are blocked. The excitation energy is instead released through radiative transitions, thereby generating strong fluorescence [26,31]. This mechanism is called RIR [27].

(2) RIV

As the family of AIE molecules continues to expand, researchers have found that the RIR model cannot fully explain the luminescence behavior of certain AIE systems without rotational units. Further computational quantum mechanic/molecular mechanic model analyses revealed that the AIE effect of these molecules stems from RIV [32]. In dilute solutions, the active vibrations of the molecular framework dissipate energy; in the aggregated state, vibrations are inhibited, and radiative transitions are restored.

### 2.3. Classical Types of AIEgens

**Figure 3:**
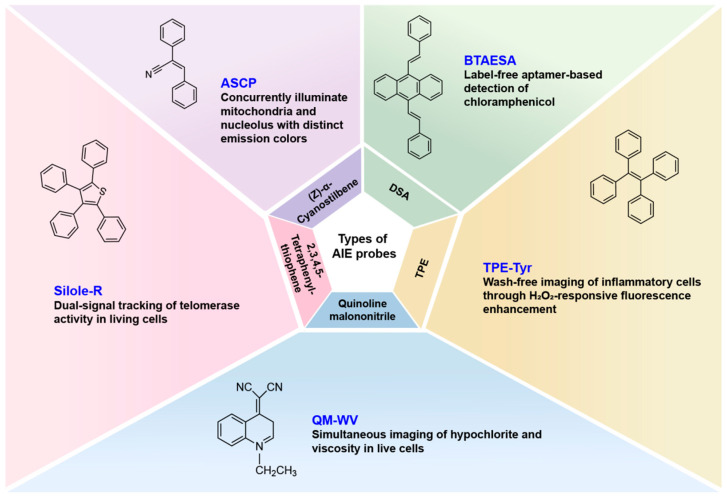
This figure presents five types of AIE probes, including their chemical structures and functions [38,39,40,41,42].

Figure 3 illustrates five representative classes of AIE probes along with their characteristic applications. The tyrosine-functionalized TPE-Tyr derivative allows wash-free imaging of the inflammatory cell via H_2_O_2_-responsive fluorescence enhancement of tetraphenylethylene (TPE) derivatives [33]. 9,10-Distyrylanthracene (DSA)-based probes like BTAESA have been used for label-free aptamer-based detection of chloramphenicol [34]. Quinoline-malononitrile scaffolds of the dual-responsive probe QM-WV allow simultaneous imaging of hypochlorite and viscosity in live cells [35]. α-Cyanostilbene derivatives, including the pyridinium compound ASCP, can concurrently illuminate mitochondria and the nucleolus with different emission colors [36]. Positively charged silole derivatives (Silole-R) have been integrated into dual-signal bioprobe systems for sensitive tracking of telomerase activity in living cells [37].

### 2.4. Advantages of AIEgens

**Figure 4:**
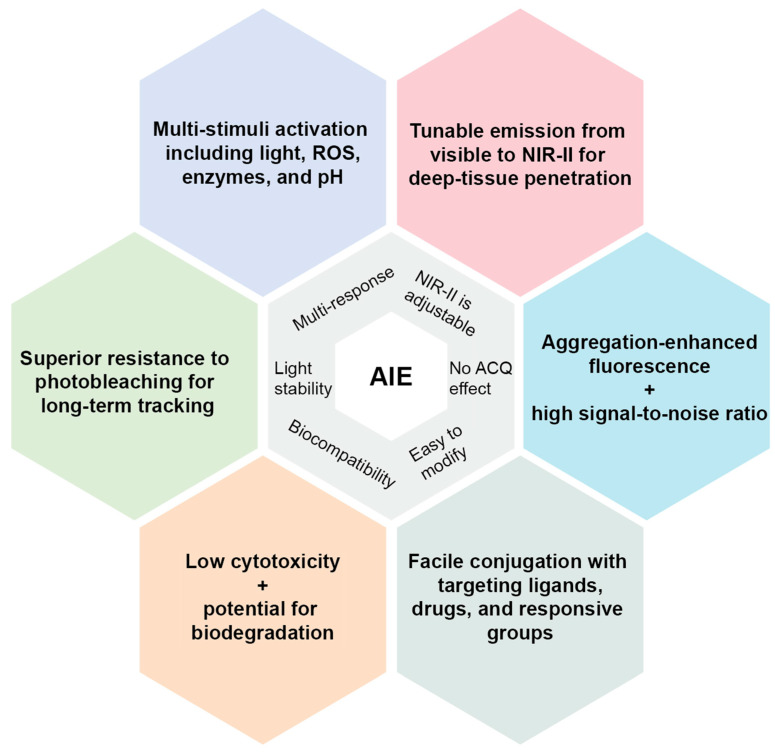
This figure illustrates six advantages of AIEgens [25,43,44].

Figure 4 summarizes six key advantages of AIEgens that make them suitable for nanotheranostic applications. First, AIEgens have aggregation-enhanced fluorescence with a high signal-to-noise ratio, so that the imaging signals are strong even when the concentration of the probe in nanocarriers or the target tissue is relatively high. Second, the emission wavelength of AIEgens can be tuned from the visible to the NIR-II region, so that the in vivo imaging has deep-tissue penetration and less background interference. Third, AIEgens are very resistant to the photobleaching, so the biological process can be long-term and repeatedly tracked without much signal decay. Fourth, AIEgens are amenable to being conjugated with the targeting ligand, the therapeutic drug, so that the multifunctional theranostic platform can be built by simple chemical modification. Fifth, AIEgens can be activated by multiple stimuli, including light, reactive oxygen species, enzymes, and pH changes, offering versatile strategies for specific diseases and therapy. Sixth, properly designed AIEgens possess low cytotoxicity and potential for biodegradation, ensuring excellent biocompatibility and favorable safety profiles for in vivo applications.

Taken together, there are multiple AIEgens with diverse molecular skeletons and functionalities, which can fulfill the need for the design and development of AIE-based nanotheranostics.

## 3. AIE-Labeled Nanotheranostics

Constructing an AIE-labeled nanotheranostic system requires three key components: AIEgen probes, nanocarrier platforms, and targeted strategies for specific diseases. In this section, we first provide an overview of the commonly used AIEgens in nanotheranostics, then classify the common types of nanocarriers used for loading and delivering these probes, and finally discuss how AIE nanotheranostics are designed based on the unique pathophysiological characteristics of different disease indications.

### 3.1. Involved AIEgens

The AIE probes investigated mainly include the following molecules. Based on the classification of AIEgen scaffolds discussed above, representative probe molecules from each category have been developed with distinct chemical modifications to fulfill specific bioimaging and biosensing functions. For instance, a DSA derivative bearing quaternary ammonium groups, (DSAC2N), has been employed as a label-free aptamer-based fluorescent probe for chloramphenicol detection in complex biological matrices [34]. In the TPE family, a tyrosine-functionalized tetraphenylethene (TPE-Tyr) exhibits H_2_O_2_-responsive AIE behavior, enabling peroxidase-mediated wash-free imaging of inflammatory cells [33]. From the quinoline-based series, a dual-responsive probe termed QM-WV, constructed on a quinoline-malononitrile scaffold, allows simultaneous monitoring of hypochlorite and viscosity changes in live HeLa cells and zebrafish [35]. Among cyanostilbene derivatives, an α-cyanostilbene pyridinium compound (ASCP) has been shown to concurrently illuminate mitochondria and the nucleolus with distinct emission colors, demonstrating the capacity of a single AIEgen for dual-color organelle imaging [36]. Furthermore, a positively charged silole derivative (Silole-R) has been integrated with a TPE-based counterpart to form a dual-signal bioprobe system capable of sensitively tracking telomerase activity both in vitro and within living cells [37].

These probes collectively possess AIE characteristics, with simple synthesis, label-free detection, no ACQ effect, high sensitivity, easy modification, high photostability, low cytotoxicity, high brightness, near-infrared emission, and good biocompatibility [45].

### 3.2. Involved Nanocarriers

Depending on the type of nanoparticle, we can regard the following three types as the mainstream for the accommodation of AIEgens.

(1) Liposomes

Liposomes are spherical vesicle structures composed of a phospholipid bilayer. The hydrophobic double layer can incorporate hydrophobic AIE probes, while the hydrophilic core can encapsulate water-soluble drugs. Long-circulating PEGylated liposomes form a hydrophilic “invisible” layer around the liposomes by connecting polyethylene glycol (PEG) chains to the phosphate head groups of the phospholipids, effectively reducing plasma protein adsorption and recognition and clearance by the reticuloendothelial system (RES) [46]. These modified liposomes are better vehicles for AIEgens. Zhang et al. designed and synthesized two AIE fluorescent probes TPE-Ma and TPE-Py with a TPE backbone, and used the biocompatible polymer DSPE-mPEG2000 as a liposomal carrier for encapsulation, successfully constructing two fluorescent nanoprobe systems, DSPE@TPE-Ma and DSPE@TPE-Py. The results showed that this nanoprobe exhibited excellent cellular permeability and biocompatibility, and outstanding photostability, and specifically targeted the lysosomes of MCF-7 breast cancer cells, demonstrating great potential in two-photon fluorescence imaging [47].

(2) Polymer nanoparticles

Polymer nanoparticles are solid colloidal particles fabricated from natural or synthetic polymers, typically ranging from 10 to 200 nm in diameter. Poly (lactic-co-glycolic acid)-polyethylene glycol (PLGA-PEG) is one of the most widely used polymer nanocarriers. Its hydrophobic PLGA core can efficiently load hydrophobic AIE probes and chemotherapy drugs, while the hydrophilic PEG shell provides long circulation characteristics. This system has three major advantages: (i) controllable synthesis, as the degradation rate can be adjusted by changing the PLGA chain length and the LA/GA ratio; (ii) a sustained-release property, with drug release lasting for several days to several weeks; (iii) easy surface functionalization, enabling active targeting by modification with targeting ligands [48]. Kim et al. prepared integrin-mediated targeted and near-infrared fluorescence (NIRF) traceable PEG-PLGA polymer nanoparticles for a combined treatment of paclitaxel (PTX) and curcumin (CUR) in breast cancer [48].

(3) Self-assembly

Self-assembled nanoparticles refer to nanostructures that form spontaneously through non-covalent interactions (such as hydrophobic forces, π–π stacking, hydrogen bonding, and electrostatic interactions) between molecular building blocks in aqueous environments. This strategy relies critically on the amphiphilic nature of the constituent molecules. By connecting the hydrophilic segments (such as PEG, peptides, and hyaluronic acid) with the hydrophobic AIE core through covalent bonds, amphiphilic AIE molecules can be constructed. These molecules can spontaneously assemble in aqueous solutions to form nanogels, achieving carrier-free delivery [49]. The advantages are: (i) Free of carrier materials: No additional nanocarriers are needed, reducing potential toxicity from additional materials. (ii) High probe density: The AIE molecules in the self-assembled system are arranged orderly, with a high-fluorescence quantum yield. (iii) Stimuli-responsive: Environment-responsive micelles such as pH, enzymes, and GSH can be designed [49]. Liang et al. designed and synthesized a two-component polymer Bio-HA(TPE-CN)-mPEG, which was constructed by introducing the hydrophobic AIE fluorescent group, acid-unstable imine bond, mPEG, and tumor-targeting ligand biotin into the main chain of hyaluronic acid. This polymer could self-assemble to form micelles and solubilize hydrophobic anticancer drugs (such as PTX). In vitro drug release studies have shown that this micelle could rapidly dissociate in acidic environments. More innovatively, this micelle emitted red fluorescence in normal physiological environments and turned blue in acidic tumor microenvironments, which could be used for real-time monitoring and quantitative drug release [49].

Table 2 compares three major types of nanomaterials relevant to AIE nanotheranostics [50].

### 3.3. Targeted Indications

There were different indications for the AIE-loaded nanotheranostic systems. According to the type of disease, the microenvironment and treatment requirements for different diseases vary, and thus different designs of AIE nanoscale diagnostic and therapeutic systems are needed. In this section, we take neoplastic, neurodegenerative and inflammatory diseases as examples.

(1) Neoplastic diseases

Tumorous diseases encompass various malignant tumors, among which lung cancer, breast cancer, colorectal cancer, prostate cancer, and liver cancer are the most common and fatal worldwide. According to data from the World Health Organization, by 2025, there will be approximately 22 million new cancer cases globally, and the number of deaths caused by cancer each year exceeds 9.7 million [51]. Among them, hepatocellular carcinoma (HCC) accounts for 75% to 85% of primary liver cancer cases [52]. Its five-year relative survival rate is only 18%, mainly due to the difficulty in early diagnosis and the lack of effective treatment methods [52]. In response to this situation, new treatment and diagnosis strategies are urgently needed to achieve early detection, real-time monitoring, and targeted intervention.

Cancer nanotheranostic technologies based on AIE are designed according to the following three main targeting strategies: (i) passive targeting utilizes the enhanced permeability and retention (EPR) effect, where the leakage of tumor blood vessels and the obstruction of lymphatic drainage enable nanoparticles to preferentially accumulate in the tumor site [16]; (ii) active targeting involves functionalization of the surface, using ligands such as folic acid, RGD peptides, and aptamers that can specifically bind to overexpressed receptors on cancer cells or tumor-related blood vessels [53]; (iii) stimuli-responsive targeting utilizes endogenous tumor microenvironment signals, including acidic pH values, elevated glutathione (GSH) levels, and excessive reactive oxygen species (ROS), or external triggering factors such as light, to achieve on-demand activation of imaging signals and drug release at the tumor site [54].

Some studies have demonstrated the feasibility and flexibility of these design methods. For example, Wu et al. developed a self-luminescent AIE photosensitizer—DB-PTZ—which can generate intense NIR-II fluorescence under 808 nanometer laser irradiation and efficiently generate reactive oxygen species (ROS), enabling tumor imaging and synergistic phototherapy in a mouse model of primary hepatocellular carcinoma (HCC) [55]. In another complementary method, Chen et al. designed an ROS and light cascade-activated NIR-II AIE detector TT-DHIn, which activates when encountering high levels of reactive oxygen species in the tumor microenvironment, generating intense NIR-II fluorescence with a high signal-to-background ratio, and further enhancing the generation of therapeutic reactive oxygen species through light irradiation [56]. The active targeting strategy also achieved good results: Kikani et al. derived a four-functional AIE gene from curcumin by introducing a phenylboronic acid group to give it pH-responsive behavior and sialic acid targeting ability; the resulting amphiphilic micelles could effectively deliver chemotherapy drugs to HepG2 liver cancer cells, and their anti-tumor effect was verified in vivo [57]. Recently, Zhu et al. reported a nanoplatform based on supramolecular metal rings, which can enhance NIR-II fluorescence for real-time tumor imaging and achieve a light thermal conversion efficiency of up to 42.7%. Moreover, the platform achieved selective drug release in glutathione-rich tumors, successfully eliminating the primary tumor in the breast cancer model and inhibiting distant metastasis [58].

(2) Neurodegenerative diseases

Neurodegenerative diseases are a group of heterogeneous disorders characterized by the gradual loss of neuronal structure and function, mainly including Alzheimer’s disease, Parkinson’s disease, Huntington’s disease, and amyotrophic lateral sclerosis. Among these diseases, Alzheimer’s disease is the most common type. As of 2025, approximately 55 million people worldwide are affected by it, and it is expected that the number of cases will triple by 2050, reaching 139 million. The medical and long-term care costs of Alzheimer’s disease and other forms of dementia are projected to reach approximately 384 billion US dollars in the United States alone by 2025 [59]. The core neuropathological features of Alzheimer’s disease include extracellular amyloid-beta (Aβ) plaques, intracellular neurofibrillary tangles composed of overphosphorylated tau protein, and chronic neuroinflammation driven by activated microglia and astrocytes. Given its significant global burden and clear pathological targets, Alzheimer’s disease has become one of the main research foci of AIE-based nanotherapy diagnostic technologies in the category of neurodegenerative diseases [60].

The AIE-based nanotherapy diagnostic technology for Alzheimer’s disease is designed based on two interrelated strategies: targeting Aβ pathology and overcoming the BBB. The first method focuses on the imaging and therapeutic intervention specific to Aβ: designing AIE probes that can specifically bind to Aβ fibrils and plaques, enabling real-time observation of amyloid protein deposition, while inhibiting Aβ aggregation, degrading existing fibrils, and clearing neurotoxic reactive oxygen species through a photodynamic mechanism. The second method addresses the severe challenge of blood–brain barrier penetration: AIE nanoplatforms can be functionalized with BBB-penetrating ligands or designed based on physical and chemical properties to promote receptor-mediated transmembrane transport or passive diffusion across the endothelial barrier [61].

These strategies have been demonstrated through recent studies. Wang et al. designed an AIE nanotherapy diagnostic system in the near-infrared II region, which can accurately monitor BBB penetration and achieve specific binding to amyloid plaques in vivo; when reactive oxygen species are triggered, the two AIE molecules encapsulated therein can be controlled to release one inhibiting the formation of Aβ fibrils and degrading existing fibrils, and the other clearing reactive oxygen species and inflammatory mediators, thereby reshaping the redox balance in the brain and achieving cognitive function improvement in Alzheimer’s disease mouse models [62]. For early diagnosis applications, Liu et al. developed an AIE fluorescent probe ZL, which can specifically bind to Aβ micelles, has high signal-to-noise ratio and excellent sensitivity, and can cross the blood–brain barrier of mice, enabling in situ imaging of Aβ fibrils in the brain [63]. In terms of expanding therapeutic dimensions, Li et al. reported a receptor-engineered AIE photosensitizer BI-TPA, which shows selective binding to Aβ aggregates, while demonstrating enhanced fluorescence brightness and reactive oxygen generation capacity, enabling targeted photooxidation of Aβ and regulation of amyloid protein aggregation to reduce neurotoxicity [64]. For further advancement in multifunctional integration, Wang et al. developed a dual-targeted NIR-II AIE nanotherapy diagnostic agent, combining two complementary AIE genes, one for Aβ-specific fluorescence imaging and micelle disruption, and the other for ROS-responsive anti-inflammatory treatment achieving synergistic BBB penetration and achieving significant cognitive recovery in Alzheimer’s disease models [65].

(3) Inflammatory diseases

Inflammatory diseases comprise a wide range of immune-mediated diseases, such as rheumatoid arthritis (RA), inflammatory bowel disease, psoriasis, systemic lupus erythematosus, and atherosclerosis. In all the above disorders, there is dysregulated immune cell infiltration and chronic inflammatory and progressive tissue destruction. RA affects about 0.5–1% of the people of the world, representing around 225 of 100,000 people in 2025 [66]. In RA there is persistent synovial inflammation, macrophage-driven immune dysregulation, and progressive joint destruction. Thus, RA is representative and clinically important for AIE-based nanotheranostic development in the big category of inflammatory diseases [67].

The AIE-based nanotheranostics for inflammatory diseases are based on three interrelated strategies. The first strategy is for immune cell-targeted imaging and immunomodulation: considering that the activated macrophages play a central role in the pathogenesis of RA, the AIE nanoprobes can be functionalized with folate or other ligands which preferentially recognize the folate receptor-β overexpressed on the pro-inflammatory M1 macrophages for targeted fluorescence imaging and the photodynamic reprogramming of macrophage polarization from the M1 to M2 phenotype [68]. The second strategy is for the ROS-responsive theranostics, taking advantage of the increased ROS of the inflamed synovial tissue to realize the site-specific fluorescence activation and on-demand drug release [69]. The third strategy is for multifunctional platforms with imaging, immunomodulation, and joint repair functions in the same nanocarrier system [68].

The practical realization of these strategies is demonstrated across a range of inflammatory conditions. In the context of RA, Wang et al. developed folate-functionalized liposomes (FA@4BC NPs) encapsulating an AIE-active curcumin-derived photosensitizer, achieving precise targeting of activated macrophages through folate receptor-β recognition; upon laser irradiation, the nanoparticles efficiently generate singlet oxygen to promote M1-to-M2 macrophage polarization and initiate anti-inflammatory signaling, with selective accumulation in inflamed joints and alleviation of cartilage damage confirmed in a collagen-induced arthritis mouse model [68]. Complementing this targeted approach, Xu et al. comprehensively reviewed AIE materials for musculoskeletal theranostics, highlighting AIEgens that target intra-articular inflammatory molecules such as ROS for early diagnosis and therapy, as well as novel AIE-containing materials developed for joint tissue repair [69]. In a recent study, Zhang et al. developed a diagnostic and therapeutic probe based on AIE, which can achieve activated NIR-II fluorescence imaging. Moreover, it can release carbon monoxide on demand in inflamed joints, achieving the synergistic effect of immune regulation and significantly alleviating the symptoms of rheumatoid arthritis [70]. Furthermore, Zhang et al. constructed a pH-responsive lipid–polymer hybrid nanoparticle system loaded with paeoniflorin for targeted macrophage polarization in a rat model of RA, illustrating how nanocarrier platforms integrating AIE imaging and immunomodulatory payloads can achieve precise therapeutic intervention at inflamed joints [71].

(4) Other diseases

The application of the AIE nanomedicine system is expanding to a wider range of disease areas, in addition to neoplastic, neurodegenerative and inflammatory diseases. Some examples are provided below.

In the field of infectious diseases, the efficient ROS production capacity of AIE photosensitizers can be used for antibacterial treatment, while its fluorescence imaging function can monitor the infection site and the wound healing process in real time. In the field of cardiovascular diseases, AIE probes targeting activated macrophages or lipid deposition in atherosclerotic plaques are under development, and they are expected to achieve the ability to provide an early warning of plaque instability and photodynamic stabilization treatment.

As discussed above, the greatest potential of the AIE nanomedicine system lies in promoting true individualized medicine. By integrating diagnostic imaging, real-time monitoring and feedback regulation, the AIE system can customize and optimize treatment plans based on the real-time images and biological information of each patient. Combined with artificial intelligence to analyze massive imaging data, it can predict treatment responses. With these superiorities, we anticipate that AIE-based nanotheranostics can be applied in the management of a variety of diseases in the near future.

## 4. Limitations and Future Directions

The above analysis has already stated that nanotheranostics have significant advantages in three areas: neoplastic diseases, neurodegenerative diseases, and inflammatory diseases. However, there are no commercially available products on the market up to now (April 2026). Evidently, some bottleneck issues hamper the clinical translation process of the relevant systems. We argue that the clinical translation of the AIE nanomedicine system still faces two core challenges:

(1) The synthesis of high-performance AIE molecules is complex, involving multiple steps of reactions, which results in low yields, difficulty in purification, and significant batch-to-batch variations [72]. For instance, the synthesis of a typical NIR-II AIE probe such as TT-DHIn involves a multistep sequence including Knoevenagel condensation, Suzuki coupling, and subsequent functional group transformations, with overall yields often below 20% and requiring rigorous chromatographic purification at each stage [73]. Such complexity inevitably introduces batch-to-batch variability in optical properties and purity, which poses a significant hurdle for standardized clinical production [72].
(2) The large-scale production of nanodelivery systems is challenging, since the methods of the laboratory are hard to scale up [74]. The difficulty of scaling up the nanocarrier fabrication can produce different particle size distributions, different drug loading efficiencies and a change in the pharmacokinetic profile when passing from the lab to the industry production. For example, studies on PLGA-PEG nanoparticles prepared by nanoprecipitation have demonstrated that increasing the batch volume from a few milliliters to several liters often results in broader polydispersity and reduced reproducibility of AIEgen encapsulation [75], which goes against the consistency of the batch needed for regulation.

In addition to the aforementioned limitations, the issue of poor biodegradability of most AIE probes also needs to be taken into consideration. As shown in the typical molecular framework in Figure 3, many AIE probes are not designed to be easily biodegradable. With the increasing concern about the long-term accumulation of synthetic molecules in organisms and the environment, this issue becomes even more important for the future clinical translation of AIE theranostics. However, in recent years, some related research on biodegradable AIE probes has been conducted; for example, Xu et al. research the creation of water-dispersible and biodegradable AIE active polymer nanoparticles [76]. These studies are overcoming this limitation.

In view of these issues, we put forward targeted answers here, which directly tackle the two abovementioned bottlenecks. For the first one, regarding the synthetic complexity of the high-performance AIE molecules, the rational molecular engineering must be first tried with the simplified synthetic route, with less reaction steps, with higher atom economy, with an easier purification procedure, of course with better yields, and with the assured batch-to-batch consistency [73]. Concurrently, the development of degradable AIE polymers can mitigate concerns regarding long-term toxicity while streamlining post-synthetic processing [72,77]. On the other hand, the development of degradable AIE polymers can alleviate the concerns about the long-term toxicity and at the same time make easier the post-synthetic process. For the difficulties in large scale of nanocarrier fabrication, the use of microfluidic technologies is a way toward achieving the manufacturing of the nanocarriers in a precise and reproducible way. The microfluidic platforms for the continuous-flow production have excellent control of the mixing kinetics and of the particle nucleation, and can then maintain the uniform particle size distributions and the consistent drug loading efficiencies even for the industrially relevant production scale [78].

In addition to these immediate technical remedies, other strategies, such as a biomimetic delivery system to avoid protein corona interference [79], the use of artificial intelligence-assisted molecular design, the regulation of standard guidelines, etc., might all contribute to expediting the clinical translation of AIE nanotheranostics.

**Figure 5:**
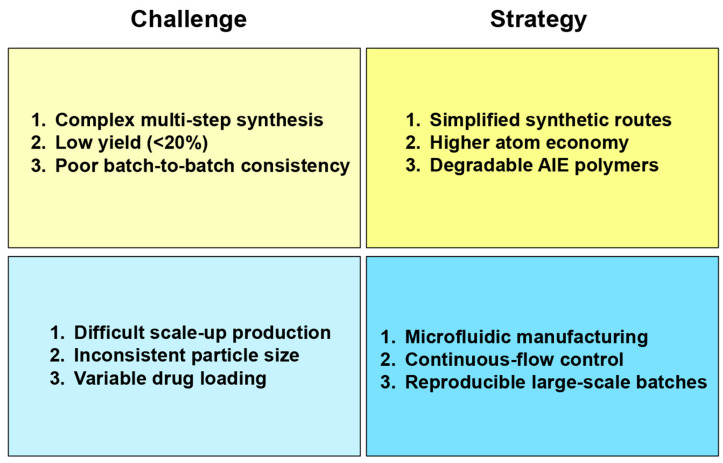
This figure illustrates the main challenges and the corresponding strategies.

The main challenges and the corresponding strategies are illustrated in Figure 5.

We think that by using rational molecular engineering to make AIEgen synthesis simpler, by developing advanced formulation technologies such as the use of microfluidics for scalable manufacturing, and by integrating artificial intelligence for predictive theranostic design, the clinical translation of AIE-based nanotheranostics will be substantially accelerated by the efforts of the interdisciplinarity. In the end, they will be the pathway toward truly personalized precision medicine where real-time imaging-guided therapy would be administered according to the individual patient’s disease profile and therapeutic response.

## 5. Conclusions

The nano-diagnosis and treatment system based on AIE embeds the optical properties of AIEgens with the multifunctional platform of nanocarrier. Therefore, it is a highly promising integrated diagnosis and treatment form for modern precision medicine.

In this review, we summarized the basic principle of AIE phenomena and the main types of probes. And we have stressed the advantages of AIE in overcoming the problem of traditional ACQ. We also list the different strategies of AIE-labeled nanomedicine systems, including the type of probe (for example, TPE and DSA), the type of nanocarrier (for example, liposomes and polymers), and the use for different diseases (for example, tumors and neurodegenerative diseases). The nanotheranostic systems based on AIE have very good performance in biological imaging, drug delivery, phototherapy and synergistic therapy.

Even though these are impressive advances, the application of nanotheranostics based on AIE technology from the laboratory to clinical practice is still faced with many obstacles, such as the synthetic complexity, the possibility of manufacturing on a large scale and the evaluation of the long-term biosafety. However, these obstacles are being slowly dealt with by the innovations in the field of molecular design and formulation engineering.

Although no clinical trials using AIE probes are underway or have been completed, we have identified a registered pilot trial (ChiCTR2400083710, launched on 1 May 2024) whose aim is to evaluate the feasibility and safety of using AIE probes for fluorescence imaging in the diagnosis of bladder cancer. It represents a crucial step towards clinical application.

In conclusion, though there are still some problems in the clinical application of AIE nanomedicine systems, with the innovation of formulation processes, especially with the method of artificial intelligence-assisted design and the idea of precision medicine, these problems are also being settled. We can look forward to AIE-labeled nanomedicine systems breaking through bottlenecks in the future and playing an important role in personalized medicine, and truly realizing the precision of health care.

## Figures and Tables

**Table 1:** Differences between ACQ and AIE.

Item	ACQ	AIE
Typical Representatives	Fluorescein, rhodamine, and azo dyes	Tetraphenylvinene (TPE), 9, 10-distyrylanthracene (DSA)
In Dilute Solution	High quantum yield	Low quantum yield
Aggregated/Solid State	Fluorescence quenching	Strong fluorescence
Mechanism	π-π stacking	Restriction of intramolecular motions
High-Concentration Applications	Limited	Advantageous

**Table 2:** Comparison of three types of nanomaterials.

Carrier Type	Drug Loading Efficiency	Stability	Biocompatibility	Clinical Translational Potential
Liposome	High	Medium	High	High
Polymer nanoparticle	High	High	High	Medium
Self-assembly	Extremely high	Low	Medium	Low

## Data Availability

No new data were created or analyzed in this study.

## Abbreviations

The following abbreviations are used in this manuscript:

AIEgen	aggregation-induced emission agent
AIE	aggregation-induced emission
ACQ	aggregation-caused quenching
MRI	magnetic resonance imaging
PET	positron emission tomography
CT	computed tomography
EPR	enhanced permeability and retention
TPE	tetraphenylvinene
DSA	9, 10-distyrylanthracene
TICT	twisted intramolecular charge transfer
RIM	restriction of intramolecular motion
RIR	restriction of intramolecular rotation
RIV	restriction of intramolecular vibration
ROS	reactive oxygen species
PEG	polyethylene glycol
RES	reticuloendothelial system
NIRF	near-infrared fluorescence
PTX	paclitaxel
CUR	curcumin
HCC	hepatocellular carcinoma
TME	tumor microenvironment
GSH	glutathione
NIR-II	near-infrared region II
AD	Alzheimer’s disease
BBB	blood–brain barrier
RA	rheumatoid arthritis
HLCT	hybridized local and charge transfer
PLGA-PEG	poly (lactic-co-glycolic acid)-polyethylene glycol

## References

[1] Modena M.M., Rühle B., Burg T.P., Wuttke S. (2019). Nanoparticle Characterization: What to Measure?. Adv. Mater..

[2] Shahzadi P. (2021). Research Anthology on Synthesis, Characterization, and Applications of Nanomaterials.

[3] Bhattacharjee S., Ershov D., Fytianos K., van der Gucht J., Alink G.M., Rietjens I.M.C.M., Marcelis A.T.M., Zuilhof H. (2012). Cytotoxicity and cellular uptake of tri-block copolymer nanoparticles with different size and surface characteristics. Part. Fibre Toxicol..

[4] Shang L., Nienhaus K., Jiang X., Yang L., Landfester K., Mailänder V., Simmet T., Nienhaus G.U. (2014). Nanoparticle interactions with live cells: Quantitative fluorescence microscopy of nanoparticle size effects. Beilstein J. Nanotechnol..

[5] Staroń A., Długosz O. (2021). Antimicrobial properties of nanoparticles in the context of advantages and potential risks of their use. J. Environ. Sci. Health Part A.

[6] Elahi N., Rizwan M. (2021). Progress and prospects of magnetic iron oxide nanoparticles in biomedical applications: A review. Artif. Organs.

[7] Karunakaran G., Sudha K.G., Ali S., Cho E.-B. (2023). Biosynthesis of Nanoparticles from Various Biological Sources and Its Biomedical Applications. Molecules.

[8] Ielo I., Rando G., Giacobello F., Sfameni S., Castellano A., Galletta M., Drommi D., Rosace G., Plutino M.R. (2021). Synthesis, Chemical–Physical Characterization, and Biomedical Applications of Functional Gold Nanoparticles: A Review. Molecules.

[9] Mura S., Couvreur P. (2012). Nanotheranostics for personalized medicine. Adv. Drug Deliv. Rev..

[10] Fares J., Davis Z.B., Rechberger J.S., Toll S.A., Schwartz J.D., Daniels D.J., Miller J.S., Khatua S. (2023). Advances in NK cell therapy for brain tumors. npj Precis. Oncol..

[11] Walsh A.J., Cook R.S., Sanders M.E., Aurisicchio L., Ciliberto G., Arteaga C.L., Skala M.C. (2014). Quantitative optical imaging of primary tumor organoid metabolism predicts drug response in breast cancer. Cancer Res..

[12] Peng X., Huang X., Du K., Liu H., Chen L. (2019). High spatiotemporal resolution and low photo-toxicity fluorescence imaging in live cells and in vivo. Biochem. Soc. Trans..

[13] Hu J.-J., Jiang W., Lou X., Xia F. (2022). Target-triggering, signal-amplified chemo/bio-sensors based on aggregation-induced emission luminogens. Cell Rep. Phys. Sci..

[14] Fahmy H.M., Bayoumi L., Helal N.F., Mohamed N.R., Emarh Y., Ahmed A.M. (2025). Emerging trends in NanoTheranostics: Integrating imaging and therapy for precision health care. Int. J. Pharm..

[15] Omidian H., Gill E.J., Cubeddu L.X. (2025). Conjugate Nanoparticles in Cancer Theranostics. J. Nanotheranostics.

[16] Deng X., Zhang Z.S., Ren T., Chen L. (2025). Regulation of oxidative stress and inflammation caused by drug accumulation in the TME based on EPR-passive strategy and active targeting. Cancer Nanotechnol..

[17] Raziq K., Xue T., Sun D.D. (2025). The shift toward nanovaccination: A comprehensive review of advancing nanovaccination for combinatory immune regulation therapies to treat infectious diseases and cancer. Int. Immunopharmacol..

[18] Aggarwal R., Magar A., Dongsar T.T., Dongsar T.S., Almoyad M.A.A., Wahab S., Goh K.W., Kesharwani P. (2026). Magnetic nanoparticles in cancer nanomedicine: Advances in targeting, hyperthermia, and theranostics. Int. J. Pharm..

[19] Raka S., Belemkar S., Bhattacharya S. (2025). Hybrid Nanoparticles for Cancer Theranostics: A Critical Review on Design, Synthesis, and Multifunctional Capabilities. Curr. Med. Chem..

[20] Zhang T., Wang B., Cheng Q., Wang Q., Zhou Q., Li L., Qu S., Sun H., Deng C., Tang Z. (2024). Polaron engineering promotes NIR-II absorption of carbon quantum dots for bioimaging and cancer therapy. Sci. Adv..

[21] Teng C., Xu Y., Wang Y., Chen D., Yin D., Yan L. (2024). J-aggregates of multi-groups cyanine dye for NIR-IIa fluorescence-guided mild photothermal therapy under 1064 nm irradiation. J. Colloid Interface Sci..

[22] Yin B., Liu X., Li Z., Ye Z., Wang Y., Yin X., Liu S., Song G., Huan S., Zhang X.-B. (2025). Ratiometric NIR-II fluorescent organic nanoprobe for imaging and monitoring tumor-activated photodynamic therapy. Chin. Chem. Lett..

[23] Zhu H.-T., Bao J.-Y., Kang J.-W., Wang A.-J., Yuan P.-X., Feng J.-J. (2024). Hydrogen-Bond-Induced Melem Assemblies to Resist Aggregation-Caused Quenching for Ultrasensitive ECL Detection of COVID-19 Antigen. Anal. Chem..

[24] Chen S.H., Xu J., Li Y., Peng B., Luo L., Feng H., Chen Z., Wang Z. (2022). Research Progress of Aggregation-Caused Quenching (ACQ) to Aggregation-Induced Emission (AIE) Transformation Based on Organic Small Molecules. Chin. J. Org. Chem..

[25] Wang W.-J., Xin Z.-Y., Su X., Hao L., Qiu Z., Li K., Luo Y., Cai X.-M., Zhang J., Alam P. (2025). Aggregation-Induced Emission Luminogens Realizing High-Contrast Bioimaging. ACS Nano.

[26] Luo J.D., Xie Z., Xie Z., Lam J.W.Y., Cheng L., Chen H., Qiu C., Kwok H.S., Zhan X., Liu Y. (2001). Aggregation-induced emission of 1-methyl-1,2,3,4,5-pentaphenylsilole. Chem. Commun..

[27] Mei J., Hong Y., Lam J.W.Y., Qin A., Tang Y., Tang B.Z. (2014). Aggregation-Induced Emission: The Whole Is More Brilliant than the Parts. Adv. Mater..

[28] Leung N.L.C., Xie N., Yuan W., Liu Y., Wu Q., Peng Q., Miao Q., Lam J.W.Y., Tang B.Z. (2014). Restriction of Intramolecular Motions: The General Mechanism behind Aggregation-Induced Emission. Chem.-A Eur. J..

[29] Zhao Z., Zhang H.K., Lam J.W.Y., Tang B.Z. (2020). Aggregation-Induced Emission: New Vistas at the Aggregate Level. Angew. Chem.-Int. Ed..

[30] Hong Y.N., Lam J.W.Y., Tang B.Z. (2009). Aggregation-induced emission: Phenomenon, mechanism and applications. Chem. Commun..

[31] Li Y.M., Zhong H.F., Huang Y.Y., Zhao R. (2019). Recent Advances in AIEgens for Metal Ion Biosensing and Bioimaging. Molecules.

[32] He Z.K., Zhao W., Lam J.W.Y., Peng Q., Ma H., Liang G., Shuai Z., Tang B.Z. (2017). White light emission from a single organic molecule with dual phosphorescence at room temperature. Nat. Commun..

[33] Cheng Y., Dai J., Sun C., Liu R., Zhai T., Lou X., Xia F. (2018). An Intracellular H_2_O_2_-Responsive AIEgen for the Peroxidase-Mediated Selective Imaging and Inhibition of Inflammatory Cells. Angew. Chem. Int. Ed. Engl..

[34] Zhang S., Ma L., Ma K., Xu B., Liu L., Tian W. (2018). Label-Free Aptamer-Based Biosensor for Specific Detection of Chloramphenicol Using AIE Probe and Graphene Oxide. ACS Omega.

[35] Huang Y., Gong Z., Wu M., Tan Z., Ding H., Ji Y., Fan C., Liu G., Pu S. (2025). A novel AIEgen fluorescent probe based on quinoline-malononitrile for monitoring and imaging ClO− and viscosity in biosystem. Spectrochim. Acta A Mol. Biomol. Spectrosc..

[36] Yu C.Y.Y., Zhang W., Kwok R.T.K., Leung C.W.T., Lam J.W.Y., Tang B.Z. (2016). A photostable AIEgen for nucleolus and mitochondria imaging with organelle-specific emission. J. Mater. Chem. B.

[37] Zhuang Y., Shang C., Lou X., Xia F. (2017). Construction of AIEgens-Based Bioprobe with Two Fluorescent Signals for Enhanced Monitor of Extracellular and Intracellular Telomerase Activity. Anal. Chem..

[38] Melo L., Silva A.M.S., Albuquerque H.M.T. (2025). The role of quinoline in the development of near-infrared fluorescent probes for diagnosis of Alzheimer’s disease. Eur. J. Med. Chem..

[39] Aizawa H., Otomo K., Honsho N., Shimazaki T., Villeneuve M., Matsuoka K., Hatano K., Terunuma D. (2012). A carbosilane dendrimer and a silacyclopentadiene analog carrying peripheral lactoses as drug-delivery systems. Bioorganic Med. Chem. Lett..

[40] Xiao Q., Duan Y., Chen Y., Liu D., Xiong W., Cheng X. (2025). Synthesis, self-assembly, photo-reactivity and application of an α-cyanostilbene pyridinium luminescent ionic liquid crystal. J. Mol. Struct..

[41] Li X., Ma K., Lu H., Xu B., Wang Z., Zhang Y., Gao Y., Yan L., Tian W. (2013). Highly sensitive determination of ssDNA and real-time sensing of nuclease activity and inhibition based on the controlled self-assembly of a 9,10-distyrylanthracene probe. Anal. Bioanal. Chem..

[42] Hariprasad V., Keremane K.S., Naik P., Babu D.D., Shivashankar S.M. (2025). Tetraphenylethylene (TPE)-Based AIE Luminogens: Recent Advances in Bioimaging Applications. Photochem.

[43] Cai X.L., Liu B. (2020). Aggregation-Induced Emission: Recent Advances in Materials and Biomedical Applications. Angew. Chem. Int. Ed..

[44] Cen P.L., Huang J., Jin C., Wang J., Wei Y., Zhang H., Tian M. (2023). Aggregation-induced emission luminogens for in vivo molecular imaging and theranostics in cancer. Aggregate.

[45] Wang S.Y., Zhou K., Lyu X., Li H., Qiu Z., Zhao Z., Tang B.Z. (2023). The Bioimaging Story of AIEgens. Chem. Biomed. Imaging.

[46] Suo M., Zhang T.F., Liang X.-J. (2025). Biomedical applications of the engineered AIEgen-lipid nanostructure in vitro and in vivo. Prog. Biomed. Eng..

[47] Zhang T.T., Huang Y., Chen X., Zheng F., Shen Y., Chen G., Ye Q., Chen K., Xiao X., Peng Y. (2024). Tetraphenylethylene-based AIE nanoprobes for labeling lysosome by two-photon imaging in living cells. Spectrochim. Acta Part A Mol. Biomol. Spectrosc..

[48] Kim K.R., You S.J., Kim H.J., Yang D.H., Chun H.J., Lee D., Khang G. (2021). Theranostic potential of biodegradable polymeric nanoparticles with paclitaxel and curcumin against breast carcinoma. Biomater. Sci..

[49] Liang N., Zhao W., Li S., Li X., Liu Z., Jiang K., Sun S. (2024). Tumor targeting pH-triggered fluorescence-switchable hyaluronic acid-based micelles with aggregation-induced emission activity for tracing drug release and intelligent drug delivery. Int. J. Biol. Macromol..

[50] Liao T.T., Chen X., Qiu F., Zhang X., Wu F., Zhao Z., Xu M., Chen M., Shen J.-W., Shen Q. (2025). Regulation of cancer-associated fibroblasts for enhanced cancer immunotherapy using advanced functional nanomedicines: An updated review. J. Nanobiotechnol..

[51] Bray F., Laversanne M., Sung H., Ferlay J., Siegel R.L., Soerjomataram I., Jemal A. (2024). Global cancer statistics 2022: GLOBOCAN estimates of incidence and mortality worldwide for 36 cancers in 185 countries. CA Cancer J. Clin..

[52] Wang J., Qiu K., Zhou S., Gan Y., Jiang K., Wang D., Wang H. (2025). Risk factors for hepatocellular carcinoma: An umbrella review of systematic review and meta-analysis. Ann. Med..

[53] Yan S., Na J., Liu X., Wu P. (2024). Different Targeting Ligands-Mediated Drug Delivery Systems for Tumor Therapy. Pharmaceutics.

[54] Wei D., Sun Y., Zhu H., Fu Q. (2023). Stimuli-Responsive Polymer-Based Nanosystems for Cancer Theranostics. ACS Nano.

[55] Wu Q., Liu Y., Zhou L., Zhao W., Wei M., Zhang Y., Han S., Umar M., He M., Tong J. (2026). DB-PTZ: A Novel NIR-II AIE Photosensitizer for Image-Guided Photothermal and Photodynamic Therapy of Hepatocellular Carcinoma. ACS Appl. Mater. Interfaces.

[56] Chen X., You Y., Lin S., Tang C., Zhu J., Liang Q., Rao D., Deng J., Ding Y., Yan D. (2026). From Spark to Flame: ROS- and Light-Cascade Activatable NIR-II AIE Probe for Precise Tumor Imaging and Self-Amplifying Phototherapy. Adv. Sci..

[57] Kikani T., Patel K., Joshi A., Gajjar D., Seshadri S., Thakore S. (2026). Design and Synthesis of an Aggregation-Induced Emission-Active Quad-Functional “Curcumin-Spiced Marvel” for Engineering of Sialic Acid-Targeted Nanoplatform for Cancer Therapy. Biomacromolecules.

[58] Chen Y., Ma W., Yu Y., Niu Q., Li Y., Yin S. (2026). AIE-Augmented NIR-II-Emissive Supramolecular Metallacycle Nanoplatform for Tumor Microenvironment-Responsive Chemo-Photothermal-Immunotherapy. Adv. Healthc. Mater..

[59] GBD 2019 Dementia Forecasting Collaborators (2022). Estimation of the global prevalence of dementia in 2019 and forecasted prevalence in 2050: An analysis for the Global Burden of Disease Study 2019. Lancet Public Health.

[60] Das S. (2026). Alzheimer’s disease basics: We all should know. Neurol. Res..

[61] Wei W., Qiu Z. (2022). Diagnostics and theranostics of central nervous system diseases based on aggregation-induced emission luminogens. Biosens. Bioelectron..

[62] Wang J., Shangguan P., Chen X., Zhong Y., Lin M., He M., Liu Y., Zhou Y., Pang X., Han L. (2024). A one-two punch targeting reactive oxygen species and fibril for rescuing Alzheimer’s disease. Nat. Commun..

[63] Liu B., Yuan L., Liu S., Zhang X., Liu F., Xin W., Wang S., Ba X., Zhang Y. (2025). An Aggregation-Induced Emission Fluorescent Probe for Detecting β-Amyloid Plaques. ChemistrySelect.

[64] Ghosh P., Mukhopadhyay S., Singh M., Ghosh S.S., Iyer P.K. (2025). Multimodal Therapeutic Approach Using Donor-Engineered AIE Photosensitizer to Combat Multifaceted Aspects of Alzheimer’s Disease. Small.

[65] Hettiarachchi S.D., Leblanc R.M. (2021). Dual targeting nano-approaches for Alzheimer’s disease etiology. Neural Regen. Res..

[66] Uke P., Maharaj A., Adebajo A. (2025). A review on the epidemiology of rheumatoid arthritis: An update and trends from current literature. Best Pract. Res. Clin. Rheumatol..

[67] Chang J.-W., Tang C.-H. (2024). The role of macrophage polarization in rheumatoid arthritis and osteoarthritis: Pathogenesis and therapeutic strategies. Int. Immunopharmacol..

[68] Wang X., Liu X., Chen J., Wu X., Xia Y., Sun L., Yang Y., Li Z., Dai H. (2025). Folate-guided AIE nanoparticles integrate macrophage-targeted fluorescence imaging and photodynamic immunomodulation in rheumatoid arthritis. Colloids Surf. B Biointerfaces.

[69] Xu H., Lin S., Hua Y. (2025). Innovations in aggregation-induced emission materials for theranostics in the musculoskeletal system. Biosens. Bioelectron..

[70] Zhang Y., Liu D., Chen W., Tao Y., Li W., Qi J. (2024). Microenvironment-Activatable Probe for Precise NIR-II Monitoring and Synergistic Immunotherapy in Rheumatoid Arthritis. Adv. Mater..

[71] Zhang J., Yang J., Yu Z., Bai H., Wang Y., Wang R. (2025). DS-Modified Paeoniflorin pH-Responsive Lipid–Polymer Hybrid Nanoparticles for Targeted Macrophage Polarization in a Rat Model of Rheumatoid Arthritis. Int. J. Nanomed..

[72] Pei Y.R., Liu L.X., Cao X.F., Zhou J., Liu C.Y. (2024). Advances in the study of AIE polymers. Front. Mater..

[73] Meng P., Han C., Scully A.D., Xiao Q., Brock A.J., Hirai T., Skidmore M., McMurtrie J.C., Chesman A.S.R., Xu J. (2021). Unconventional, Gram-Scale Synthesis of a Molecular Dimer Organic Luminogen with Aggregation-Induced Emission. ACS Appl. Mater. Interfaces.

[74] Xu X.Y., Chen L., Yang L., Li B., Kim Y., Yoon C., Yoo J., Wang L., Chen S., Gu M. (2025). Top-down bioinspired nanotheranostics with AIE luminogens. Coord. Chem. Rev..

[75] Sagoe P.N.K., Velázquez E.J.M., Espiritusanto Y.M., Gilbert A., Orado T., Wang Q., Jain E. (2023). Fabrication of PEG-PLGA Microparticles with Tunable Sizes for Controlled Drug Release Application. Molecules.

[76] Xu D.Z., Liu M., Zou H., Tian J., Huang H., Wan Q., Dai Y., Wen Y., Zhang X., Wei Y. (2017). A new strategy for fabrication of water dispersible and biodegradable fluorescent organic nanoparticles with AIE and ESIPT characteristics and their utilization for bioimaging. Talanta.

[77] Huang S.Q., Wang Z., Lu X., Gong J., Cheng L., Wang J., Zhang J., Alam P., Lv Z., Zhang H. (2024). Interception and in Situ Eradication of Airborne Pathogens by Ecofriendly, Biodegradable Wooden Filters. ACS Mater. Lett..

[78] Huang C.Y., Chen M., Du L., Xiang J., Jiang D., Liu W. (2022). Microfluidic Synthesis of the Tumor Microenvironment-Responsive Nanosystem for Type-I Photodynamic Therapy. Molecules.

[79] Dave R., Pandey K., Khatri V., Patel R., Gour N., Bhatia D. (2025). Biological AIE Molecules: Innovations in Synthetic Design and AI-Driven Discovery. Adv. Biol..

